# An Immunosensor Based on Au-Ag Bimetallic NPs Patterned on a Thermal Resistant Flexible Polymer Substrate for In-Vitro Protein Detection

**DOI:** 10.3390/polym11081257

**Published:** 2019-07-29

**Authors:** Pan Wang, Shiliang Wei, Lifen Tong, Xiaohong He, Yun Bai, Kun Jia, Xiaobo Liu

**Affiliations:** Research Branch of Advanced Functional Materials, School of Materials and Energy, University of Electronic Science and Technology of China, Chengdu 610054, China

**Keywords:** LSPR nanosensor, bimetallic nanoparticles, in-vitro protein detection, thermal annealing, flexible substrate

## Abstract

Nanosensors based on flexible polymers have emerged as powerful tools for next generation smart devices in the recent years. Here, we report a facile protocol to fabricate an immunosensor supported by a thermally resistant flexible polymer substrate (polyarylene ether nitrile, PEN). The immunosensor is a localized surface plasmon resonance (LSPR) optical sensor for in-vitro protein detection based on anti-body coated gold-silver bimetallic nanoparticles (Au-Ag NPs) immobilized on a PEN substrate. Plasmonic spectroscopy and morphological characterization show that the Au-Ag NPs essentially exhibit a more uniform size distribution and higher quality factors than those from single-component Au NPs. Furthermore, it should be noted that the robust PEN substrate in this nanosensor acts a flexible substrate to support Au-Ag NPs and immobilize the nanoparticles via quick thermal annealing at 290 °C. Thanks to these merits, a prostate-specific antigen (PSA) concentration as low as 1 ng/mL can be specifically discriminated via the prepared PEN/Au-Au NPs, which confirms that the protocol reported in this work can be readily adapted for the construction of various flexible immunosensors for different applications.

## 1. Introduction

Nowadays, flexible substrates bring significant benefits to the manufacture of various high-tech products [1,2]. Polymeric substrates have, especially, been widely used in flexible solar cells [3], displays [4], stretchable devices [5], wearable health monitoring technology [6], and optical sensors [7,8]. Compared to common solid substrates, flexible substrates have the advantages of versatile processability, good mechanical properties, and abundant functionalization strategies. Although a range of transparent flexible polymer substrates have already been used in various optical devices, it is still a great challenge for polymer substrates to achieve a balance between optical function and mechanical/thermal properties. This will inevitably hamper the wider development of flexible optical devices since their fabrication normally involves higher temperature or aggressive conditions. In this sense, polyarylene ether nitrile (PEN) is a type of high-performance engineering thermoplastic exhibiting outstanding mechanical/thermal properties [9,10] and rich functionalization (i.e., visible light transmittance, intrinsic fluorescence, biocompatibility, etc.) [11], could be employed as an acceptable substrate for the construction of various high performance flexible devices combined with mechanical, thermal and optical properties [12,13].

In recent decades, noble metallic nanoparticles have been the subject of increased research interests in many fields including catalysis, medicine, and electronics owing to their outstanding optical, catalytic and electrical properties [14,15,16]. Noble metal nanoparticles exhibit a unique optical effect called localized surface plasmon resonance (LSPR), which is derived from the collective oscillation of their free electrons upon incident light [17,18]. According to Mie’s theory, the LSPR property is strongly dependent on the morphology of the particles, the spacing between the particles and the surface dielectric environment [19], which lays the foundation for the application of noble metal nanoparticles in various nanosensors. Generally, the metallic nanoparticles have been fabricated on substrates, mainly via inkjet printing [20,21], self-assembly of metal precursors [22], nanomanipulation methods [23], and thermal annealing [24]. In particular, thermal annealing has become a promising protocol in nanosensor fabrication because of its facile fabrication steps, green process, excellent stability, and scale preparation potential [25]. The mechanism of this method is to use a substrate material that will soften above its glass transition temperature (*T_g_*), while the continuous metal film on the top will aggregate to form nanoparticles and become embedded on the surface of the underlying substrate [26]. Specifically, different noble metals (e.g., gold, silver, and gold-silver alloy) have been immobilized on various rigid substrates (e.g., glass, silicon, TiO_2_) to fabricate nanosensors for different applications via thermal annealing [24,27,28,29]. In our previous work, different sized gold nanoparticles (Au NPs) on a flexible PEN substrate had been obtained via vacuum evaporation combined with thermal annealing for the first time [13]. Because the *T_g_* of PEN is just about 200 °C, the annealing temperature was greatly reduced compared to that of the general glass substrate. Meanwhile, the flexible PEN substrates also can endow the prepared metal NPs/polymer composite with versatile processability, good mechanical properties, and abundant functionalization strategies. However, a relatively long annealing time (~2 h) was required to obtain Au NPs, and their LSPR peaks normally exhibited larger full width at half maximum (FWHM) values, which implied that the flexible plasmonic substrate still needs to be greatly improved for practical sensing applications.

It is well-known that gold-silver bimetallic nanoparticles (Au-Ag NPs) show evidently improved optical properties compared to those of single-component Au or Ag NPs [30,31,32]. For instance, the Au-Ag NPs combined with the high refractive index of the Ag component and facile biofunctionalized Au component exhibit an ultrasensitive performance in immunodetection [33]. Meanwhile, the characteristic LSPR peak wavelength of Au-Ag bimetallic NPs can be tuned to a much wider range than that of pure Au NPs (~520 nm) and Ag NPs (~400 nm) [34,35]. Consequently, we herein evaporated an Ag layer on top of the thin Au layer on the PEN substrate to improve the quality factor of the flexible plasmonic substrate. In addition, we discovered that a rapid annealin treatment of the plasmonic substrate is able to create stable and uniform Au-Ag NPs on PEN substrate, demonstrating a higher quality LSPR peak, which contributed to the fabrication of immunosensors based on a flexible polymer substrate for the sensitive and selective in-vitro detection of prostate specific antigen (PSA).

## 2. Experimental Section

### 2.1. Materials

The PEN used in this work was synthesized in our laboratory though the synthetic procedure reported in the previous report [36]. *N,N*-dimethylacetamide (DMAc) was purchased form Kelong Chemicals (Chengdu, China). Gold and silver target materials were obtained from Zijin Mining group Co. Ltd. (Xiamen, China) *N*-(3-Dimethylaminopropyl)-*N*’-Ethylcarbodiimide Hydrochloride (EDC), *N*-Hydroxysuccinimide (NHS), and 11-mercaptoundecanoic acid (MUA) were purchased from J&K Chemicals (Beijing, China). The anti-PSA, prostate-specific antigen (PSA), and albumin from bovine serum albumin (BSA) prepared in phosphate buffered solution (PBS, pH = 7.4) were purchased from Shanghai Linc-Bio Science (Shanghai, China).

### 2.2. Preparation of PEN Casting Film

The PEN film was prepared using the classical casting method. Typically, 1 g of PEN was dissolved in 11 mL DMAc solvent under magnetic stirring to form a homogeneous solution. Then the obtained solution was casted onto the horizontal glass plate. The transparent and tough polymer film was formed after a heating process at temperatures of 80, 100, 120, 140 and 160 °C (taking 2 h for each step). Afterward, the obtained PEN film was cut into small pieces (1.4 × 1.4 cm) and washed with ethanol before further processes.

### 2.3. Preparation of Au-Ag NPs Patterns on PEN Substrates

In order to create a well-defined Au-Ag NPs pattern, the conventional sample holder for transmission electron microscopy was fixed onto the PEN substrate, which was subjected to metal evaporation in a high vacuum evaporator. All the metallic films were evaporated under a slow deposition rate (0.01 nm/s) and high vacuum (<1 × 10^−6^ Torr). The pure gold films with a nominal thickness of 2 nm were evaporated using a gold target, while the gold-silver films were fabricated by evaporating silver on the pre-formed gold film. Afterward, the as-evaporated metallic-PEN films underwent rapid annealed under different temperatures (i.e., 250, 270, 290 and 310 °C) in a nitrogen atmosphere with a heating rate of 30 °C/min starting from room temperature. The temperature was maintained for 2 min, then they were naturally cooled to room temperature.

### 2.4. Preparation of Optical Probes for Specific Immunodetection of Prostate Specific Antigen

The as-evaporated samples were immersed in EtOH for 30 min at room temperature to remove the unstable metallic nanoparticles. Then, the stable metallic nanoparticles were modified with 11-mercaptoundecanoic acid (MUA, 1 mM) in an EtOH solvent at room temperature overnight to obtain the thiolated plasmonic surfaces. After washing with ethanol, the terminal carboxylic groups of thiolated Au-Ag NPs were activated with an aqueous mixture solution of EDC and NHS (0.4 mM/0.1 mM) for 50 min at room temperature. This was followed by washing with deionized water. Furthermore, the PSA antibody (anti-PSA, 100 ng/mL) was incubated with the active thiolated metallic nanoparticles for 5 h at 4 °C. After washing the samples with phosphate buffered saline (PBS) buffer and deionized water, the biofunctionalized samples were incubated with the PSA antigen at different concentrations for 2 h at room temperature. Finally, the antigen modified samples were also washed with PBS buffer and deionized water, followed by drying in a vacuum oven for each step. They were then used for recording the specific LSPR peak.

### 2.5. Thermo Analysis, Molecular Analysis, Alloy Structure, Location Spectroscopy, and Morphology Characterization

The results of the glass transition temperature (*T_g_*) and gel permeation chromatography (GPC) analysis of PEN were recorded on a differential scanning calorimetry instrument (DSC Q100, TA Instrument, New Castle, PA, USA) and a PL-GPC220 system (Agilent Technologies, Palo Alto, USA) using polystyrene as the standard and THF as eluent, respectively. The LSPR spectra were recorded on a customized microscope (BA410E, Motic, Hongkong, China) system equipped with a portable spectrometer (NOVA, Ideaoptics Instrument, Shanghai, China), optical fiber (FIB-M-600-NIR, Ideaoptics Instrument, Shanghai, China), and analysis software (Morpho, Ideaoptics Instrument, Shanghai, China). In particular, the detection area of the microscope was adjusted to match the single square of Au-Ag NPs patterns to ensure the reproducibility of the LSPR peak evolution during different bio-functionalization steps. The low-magnification microphotographs were also taken on the same microscope which was equipped with a camera (DigiRetina16, Tucsen Photonics, Fuzhou, China). A scanning electron microscope (JSM-5900LV, Jeol, Akishima, Japan) was used to characterize the surface morphology of bimetallic NPs. X-ray photoelectron spectroscopy (XPS) was measured on an ESCA 2000 (VG Microtech, Uckfield, UK) employing a monochromic Al Ka source.

## 3. Results and Discussion

**Scheme 1:**
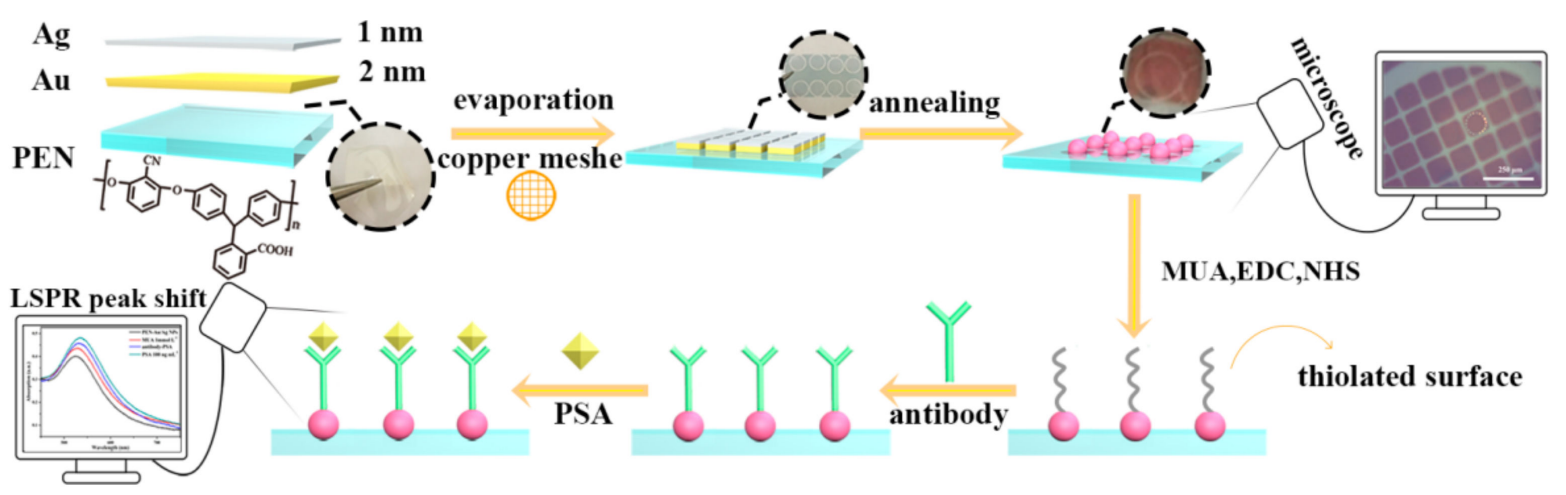
Schematic illustration for the fabrication of Au-Ag bimetallic nanoparticles (Au-Ag NPs) on a polyarylene ether nitrile (PEN) flexible substrate, and the bio-functionalization for specific immunodetection.

Polyarylene ether nitrile is a type of high-performance thermoplastic which is generally used as a robust matrix for the fabrication of advanced engineering materials. As shown in Scheme 1, the PEN employed in this article contains a phenolphthalin moiety in its backbone, which contributes to its good thermal stability and amorphous nature [9]. Moreover, the weight-average molecular weight (*M_w_*), number-average molecular weight (*M_n_*) and polydispersity index (*M_w_*/*M_n_*) measured by GPC were 98276 g/mol, 64107 g/mol, and 1.53, respectively. Therefore, the casted PEN substrate exhibited good mechanical properties (i.e., a flexible strength of ~80 MPa), thermal stability (i.e., *T_g_* around 230 °C), and a high visible light transmittance. After depositing an ultrathin metallic film on the flexible PEN substrate, the Au-Ag bimetallic NPs could be fabricated only after annealing the samples above 200 °C according to our previous work. In addition, the reproducibility of the LSPR peak of formulated Au-Ag NPs during this process cannot be ensured unless the same area was probed for each measurement. Therefore, we employed the conventional TEM sample holder to divide continuous Au-Ag NPs surfaces to well-organized micro-meter scale patterns. These patterns have the exact same size as the optical fiber of the microscope, as highlighted in the upper-right microscope image of Scheme 1. The independent image is displayed in the supporting information as Figure S1 which showed the test area located by aureole in detail. Thus LSPR spectra of the same area couldbe recorded for each step including evaporation, annealing and biofunctionalization.

### 3.1. Influence of an Additional Ag Layer

The evaporation thickness and annealing temperature are essential parameters to determine the morphology and the LSPR spectra of Au NPs on a PEN substrate. The optimized thickness of the Au film is 2 nm according to our previous work, but a much longer annealing time up to 2 h was required to promote the transformation of a semi-continuous metallic film into Au NPs with an inhomogeneous size distribution. Given the beneficial size-shaping capacity of silver and the well-known principle of time-temperature equivalency [37], we introduced an additional silver layer on top of the Au film and annealed the bimetallic film evaporated sample at a much higher temperature (250–310 °C) for a shorter time (2 min).

**Figure 1:**
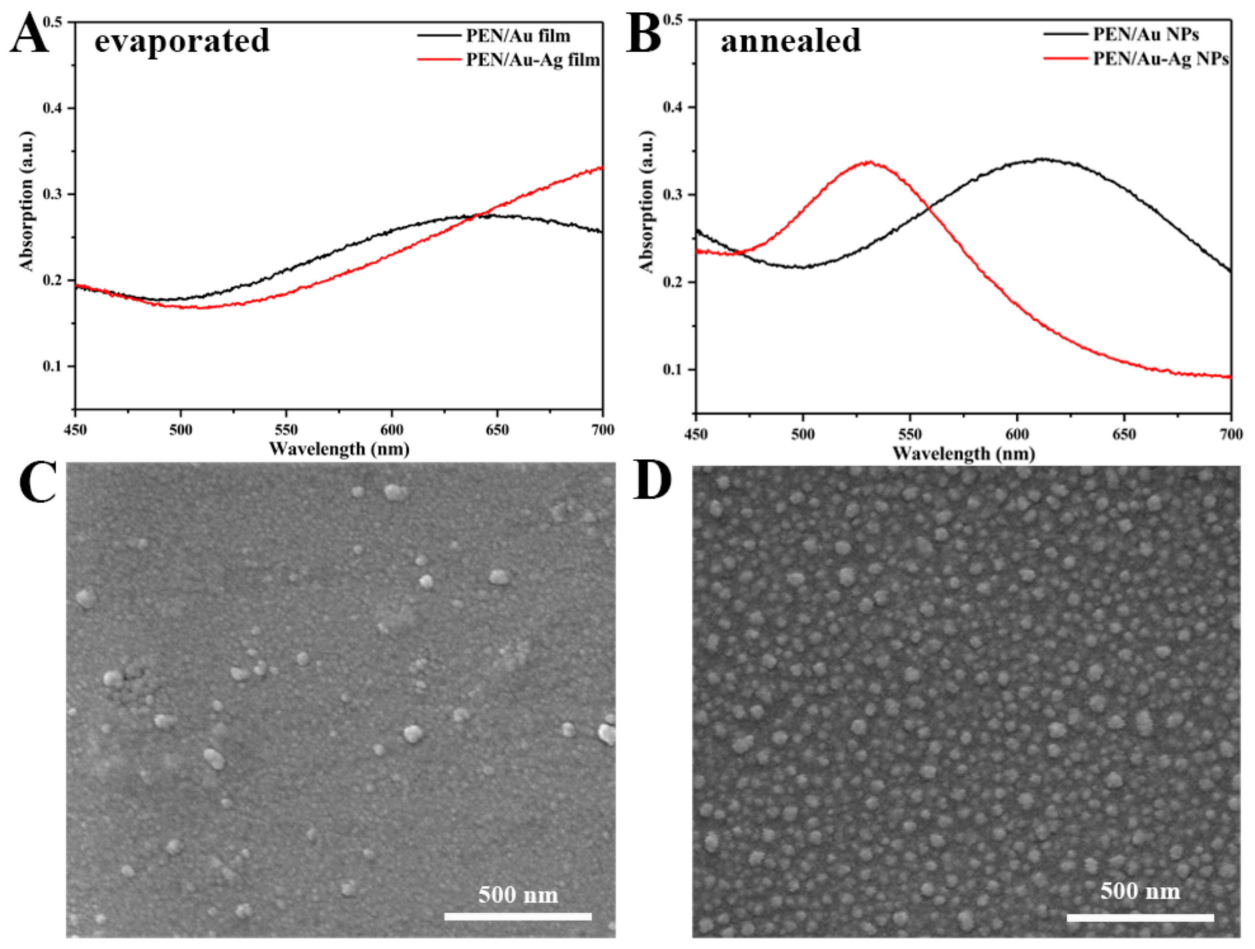
The localized surface plasmon resonance (LSPR) spectra of single metallic PEN/Au and bimetallic PEN/Au-Ag samples before (**A**) and after thermal annealing (**B**). Their corresponding SEM images show the surface morphology of Au NPs (**C**) and Au-Ag NPs (**D**) after annealing. Both samples were annealed at 290 °C.

As shown in Figure 1A, the as-evaporated PEN/Au sample shows a very broad LSPR band, which is owing to the formation of discrete Au particles resulting from an ultrathin gold deposit on the PEN substrate which is similar to what was found in our previous work. Moreover, the PEN/Au-Ag bimetallic sample displays no absorption peak as a typical feature of a continuous metallic film (see Figure 1B). Because of the spectrum of the bimetallic Au-Ag sample, the discrete surface of the metallic film was modified by the addition silver layer. After annealing, the spectra of both samples show a typical specific band of plasmonic nanoparticles (Figure 1A,B). The LSPR peak wavelengths of Au NPs and Au-Ag NPs on the PEN substrate after annealing were detected at 613 and 532 nm, respectively. In addition, it is evident that the FWHM of Au NPs (162 nm) is much wider than that of the Au-Ag NPs (118 nm), which implies that the bimetallic Au-Ag NPs should have a more uniform size distribution. As shown in Figure 1C, the inhomogeneous Au NPs were situated on the surface of the PEN substrate, while spherical nanoparticles with uniform morphology were detected in Au-Ag NPs sample, according to Figure 1D. Based on the LSPR spectra and the surface morphology characterization results, we confirmed that the Au-Ag NPs exhibited improved LSPR properties compared to the AuNPs.

**Figure 2:**
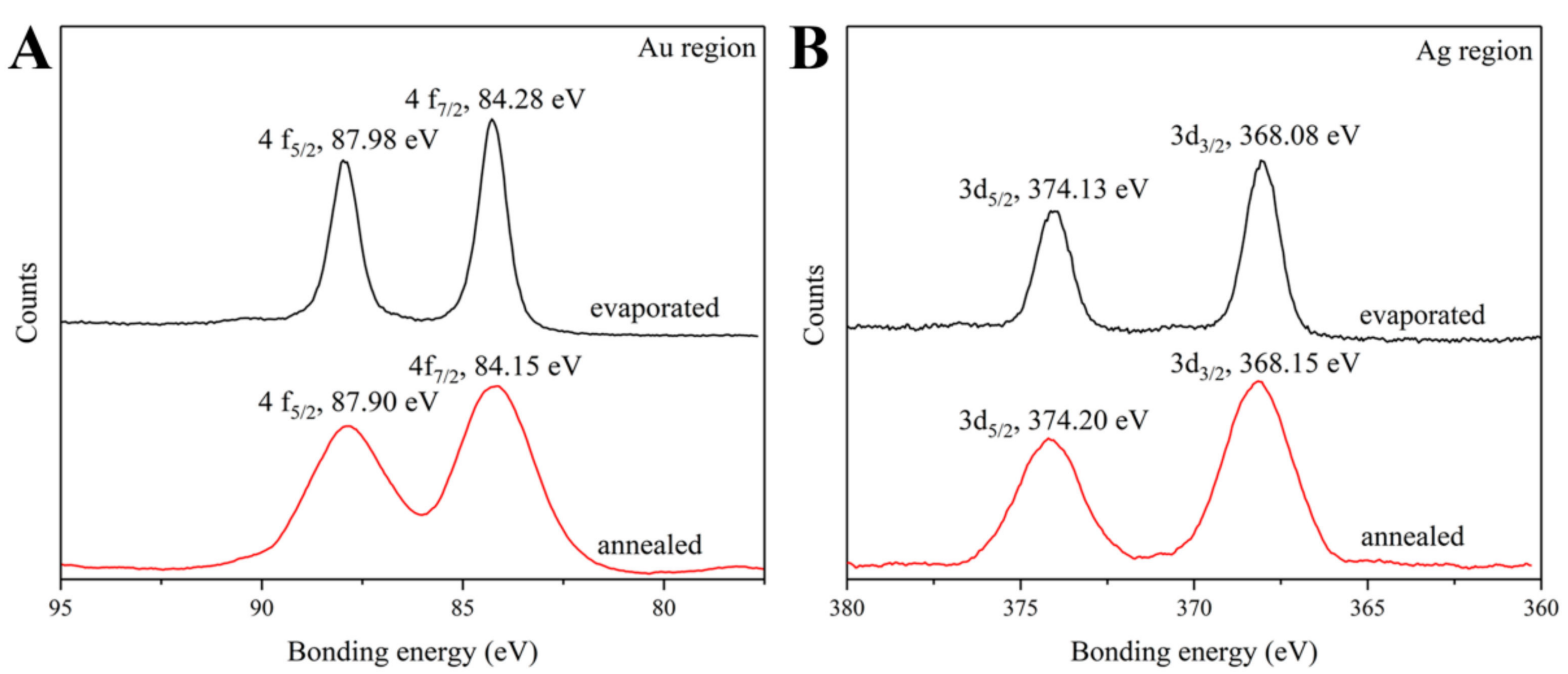
The X-ray photoelectron spectroscopy (XPS) spectra of Au (**A**) and Ag (**B**) regions from both the evaporated (black line) and annealed (red line) samples.

The presence of gold and silver on the surface of the evaporated sample and annealed sample were measured using the XPS technique. For the evaporated sample, as shown in Figure 2A,B, the two peaks located at 84.28 and 87.98 eV belonged to 4f_7/2_ and 4f_5/2_, respectively, of the Au atom. The binding energy corresponding to 3d_5/2_ and 3d_3/2_ of Ag was detected at 374.20 eV and 368.15 eV, respectively. Moreover, the binding energy for both Au and Ag remained unchanged for the evaporated and thermal annealed samples, which implies that the rapid annealing driven plasmonic nanoparticle formation is a physical process that doesn’t involve any chemical interaction with the PEN substrate. It was found that the mole percentages of Au and Ag were recorded as 66.14% and 33.86% in the evaporated sample, respectively, while their percentage changed to 62.10% for Au and 37.90% for Ag in the annealed sample. The increased relative surface concentration of silver after thermal annealing may be attributed to the migration of silver onto the top surface of the nanoparticles during the thermal treatment [38].

### 3.2. Influence of Rapid Annealing Temperature on Au-Ag NPs

It is well-known that the plasmonic properties and morphology of metallic NPs are strongly dependent on the annealing temperature, and the annealing temperature for nanoparticle formation should be decided based on the following three reasons: (i) Generally, the transformation from semi-continuous gold film to discrete nanoparticles can be activated via thermal annealing above the melting point at around 200 °C. The so-called “solid-state dewetting” [39], process is dedicated to minimizing the overall surface energy of the composite system. (ii) The annealing temperature should be higher than the glass transition temperature (230 °C, see in Figure S2) of the polymer substrate, which facilitates the partial embedding and consolidation of plasmonic nanoparticles into the underlying polymer substrate. (iii) Higher annealing temperatures can be used to reduce the annealing time according to the basic principle of time-temperature equivalency. Therefore, the annealing temperature ranging (ranging from 250 to 310 °C) was investigated in this work to modulate the Au-Ag NPs morphology and plasmonic properties.

**Figure 3:**
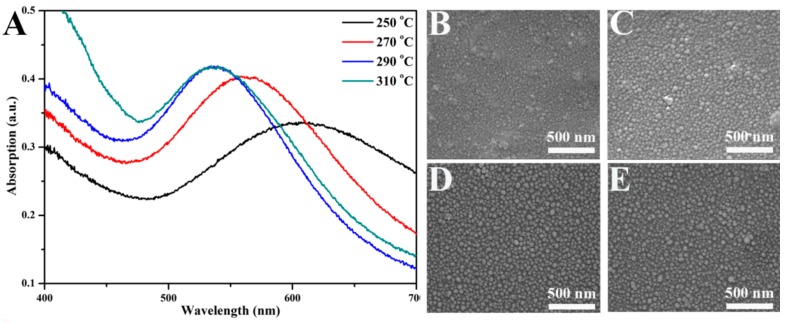
The LSPR spectra of the PEN/Au-Ag NPs after annealing at different temperatures (**A**). The corresponding SEM results of the PEN/Au-Ag NPs undergoing annealing at 250 °C (**B**), 270 °C (**C**), 290 °C (**D**), and 310 °C (**E**).

It can be seen from Figure 3A that as the annealing temperature increased from 250 to 290 °C the typical LSPR wavelength of the annealed sample was steadily blue-shifted from 610 to 535 nm, and the maximum absorption was gradually increased. Further increasing of the annealing temperature up to 310 °C did not bring about the expected modulation of the plasmonic wavelength but, instead, resulted in the enhancement of the overall absorption band from the deep-blue range, which can be partially attributed to the chemical transformation of the underlying polymer substrate during high temperature annealing. In addition, the surface morphology of Au-Ag NPs annealed at different temperatures is shown in Figure 3B,E. It is clear that both the size and interparticle spacing of Au-Ag NPs was increased as the annealing temperature was increased from 250 (Figure 3B) to 290 °C (Figure 3D), while the sample treated at the highest temperature of 310 °C exhibited similar sizes but a slightly inhomogeneous size distribution (Figure 3E) when compared to those of the 290 °C annealed sample. In summary, the thermal annealing temperature range for the creation of Au-Ag NPs on a PEN substrate is located at 250 to 310 °C, while the optimized annealing temperature to fabricate well-organized and uniform Au-Ag NPs is 290 °C, and in this case, a narrow size distribution with an average diameter of 35 nm was recorded according to Figure S3. Moreover, the stability of the PEN/Au-Ag NPs has also been further measured by immersing them in absolute ethanol and recording the intensity of the absorption peak after immersion in absolute ethanol for different lengths of time. The result illustrated in Figure S4 indicates that Au-Ag NPs are indeed embedded inside the underlying PEN substrate and possess good stability.

### 3.3. Immunodetectionof the PSA Antigen Based on a PEN/Au-Ag NPs LSPR Sensor

**Figure 4:**
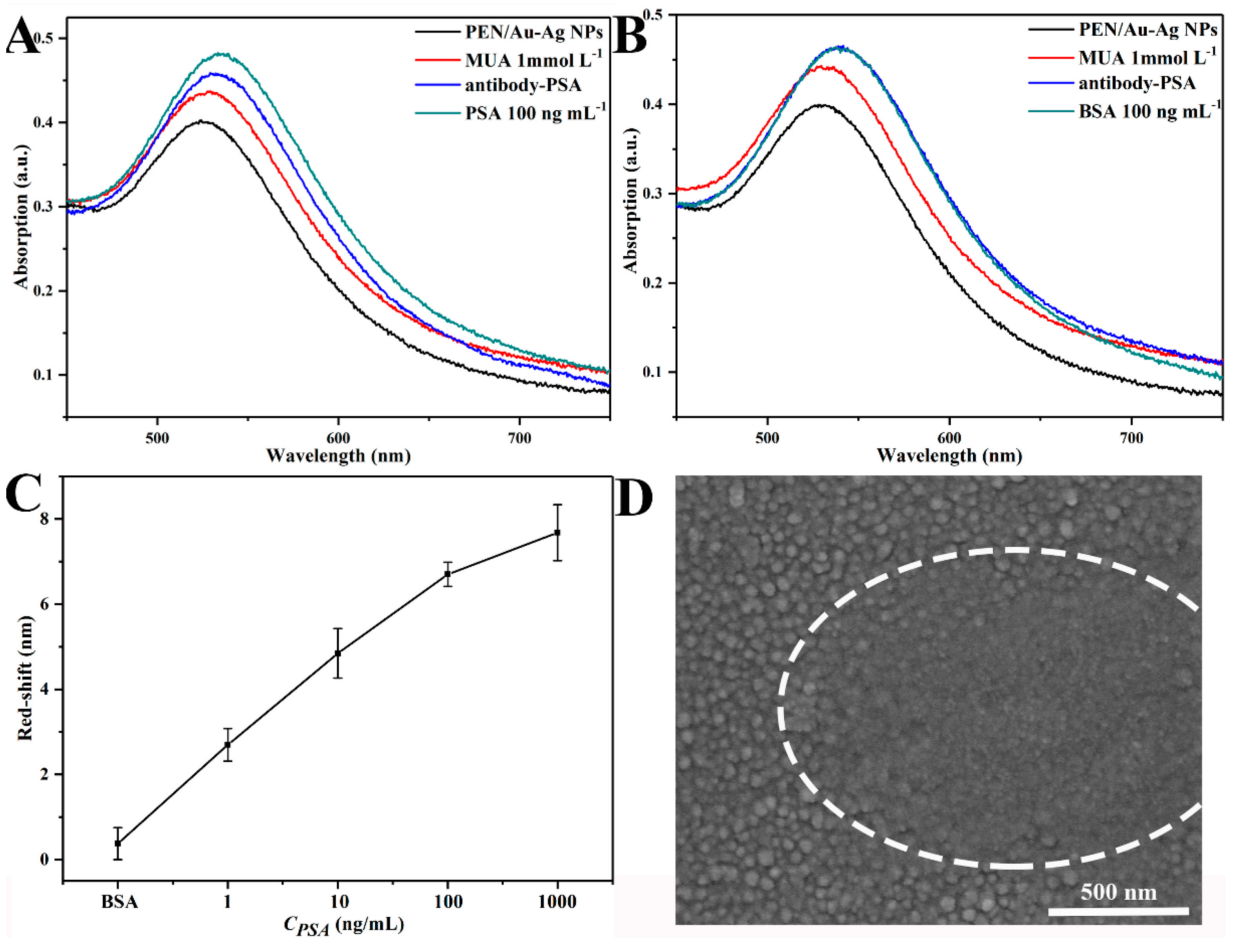
The LSPR spectra evolution of PEN/Au-Ag NPs upon specific immunodetection of prostate-specific antigen (PSA) (**A**). Negative control experiment results from using non-specific bovine serum albumin (BSA) (**B**). Dose dependent responses curve to different concentrations of PSA (**C**), and SEM images of PEN/Au-Ag NPs after specific binding of 100 ng/mL PSA (**D**).

In addition to the selection of optimized plasmonic substrates, a specific antibody needs to be immobilized onto the optimized bimetallic Au-Ag NPs to enable immunodetection of the antigen. Considering that several steps are involved in the construction of immunosensors, the LSPR spectra should thus be collected from the exact same area for each step to ensure reproducibility and measurement precision. For this reason, we employed the conventional cooper sample holder for TEM characterization as a mask during the evaporation process to fabricate Au-Ag NPs patterns that have more or less the same area (110 × 110 μm) as those of the microscope detection zone (see Scheme 1). With the assistance of these well-organized micro-patterns, the LSPR spectra for each biomodification of Au-Ag NPs, and their responses to specific antigen PSA and the negative control of BSA, were recorded in Figure 4A,B, respectively. Since the samples were immobilized with the anti-PSA, the PSA could specifically bind to it and, therefore, changes were observed in the spectrum. On the contrary, the BSA albumin, which is non-specific to the anti-PSA, did not show any change in the spectra [27]. Therefore, the LSPR spectra of the positive control experiment were steadily red-shifted, along with experiencing MUA thiolation, antibody immobilization, and antigen binding. However, there was no additional wavelength shift after exposure to the non-specific BSA in the negative control experiment, which unambiguously confirmed that the fabricated Au-Ag NPs on the PEN substrate can be constructed as a flexible specific immunosensor for PSA detection.

In addition, the dose-dependent response for the PSA in different concentrations is illustrated in Figure 4C, where the different concentrations of the PSA ranging from 1 to 1000 ng/mL showed a trend in which the degree of shift in wavelength increased with increasing PSA concentrations. However, the narrow range of the red-shift changed from ~2 nm to ~8 nm as the concentration of the PSA increased from1 to 1000 ng/mL. Moreover, as shown in Figure S5, the dose dependency ranged from 1 to 10 ng/mL, showed large error bars, a poor linear correlation coefficient (R^2^ = 0.90), and a limit of detection (LOD) calculated to be 3.08 ng/mL. Therefore we think this prepared immunosensor basically provide semi-quantitative results for the moment. Moreover, the PSA level for healthy people is below 4 ng/mL, and PSA higher than 10 ng/mL is a diagnostic indicator for prostate cancer. In our system, the LOD and linear range results could satisfy the PSA detection in prostate cancer patients (4 to10 ng/mL) in early diagnosis and is also comparable to some recently published works based on the LSPR absorption peak shift [27,40]. This indicates that the present PEN/Au-Ag NPs has the potential to be a PSA specific immunodetection material. In order to achieve the quantitative results with high sensitivity, additional signal amplification strategies such as modulation of nanostructures morphology, enhancing the coupling effects between individual plasmonic transducer, constructing sandwich like immunocomplex, etc. should be combined with the current system. These would be more appropriate topics for subsequent works dedicated to analytical improvements. Finally, the surface morphology of the Au-Ag NPs after specific binding of 100 ng/mL of PSA was characterized using SEM to show the change. It was found that the Au-Ag NPs were evidently covered by a top protein layer (highlighted by the white circle). Different from the noble metal NPs fabricated on an untreated glass substrate [41], it should be noted that the proteins could also be detected from the interstitial area between the nanoparticles, according to Figure 4D. This should be attributed to the presence of surface carboxyl groups of the PEN substrate, leading to enhanced immobilized antibody density.

## 4. Conclusions

To summarize, Au-Ag bimetallic nanoparticles with a uniform size distribution and narrow LSPR spectra have been fabricated on a flexible robust PEN substrate via vacuum evaporation combined rapid annealing protocol. It was found that the incorporation of a 1 nm thick additional silver layer on top of the 2 nm gold film contributed to the preparation of ~35 nm sized Au-Ag NPs with greatly improved optical properties after rapid annealing at 290 °C. Subsequently, a high density of an anti-PSA antibody was immobilized on the Au-Ag NPs decorated carboxylated PEN substrate to construct an immunosensor for PSA detection. Finally, the fabricated immunosensor was able to specifically detect, in vitro, concentrations of PSA protein as low as 1 ng/mL based on the control experimental results and dose-dependent responses. Although the in-vitro protein detection performance requiers further improvement, this work reveals a facile way to fabricate immunosensors on a massive scale using an optically transparent and thermally resistant flexible polymer substrate, which can enchance the prepared immunosensors’ stability, machinablility and so on. Meanwhile, the backbone chemical structure of high performance PEN can be further modified to activate additional optical properties including fluorescence emission, surface enhanced Raman spectroscopy, and liquid crystal transition, which would largely expand the design strategies for constructing multifunctional flexible nanosensors in the future.

## Supplementary Materials

The following are available online at https://www.mdpi.com/2073-4360/11/8/1257/s1.
