# Supplementary material for: An Immunosensor Based on Au-Ag Bimetallic NPs Patterned on a Thermal Resistant Flexible Polymer Substrate for In-Vitro Protein Detection
Source: Polymers (Basel). 2019 Jul 29;11(8):1257. doi: 10.3390/polym11081257 (PMC6724069; doi:10.3390/polym11081257)
Supplement: Supplementary file 1 [file polymers-11-01257-s001.pdf]

An Immunosensor Based on Au-Ag Bimetallic NPs Patterned on a Thermal Resistant Flexible Polymer Substrate for In-vitro Protein Detection

Pan Wang, Shiliang Wei, Lifen Tong, Xiaohong He, Yun Bai, Kun Jia *, Xiaobo Liu *

Research Branch of Advanced Functional Materials, School of Materials and Energy, University of Electronic Science and Technology of China, Chengdu 610054, P. R. China

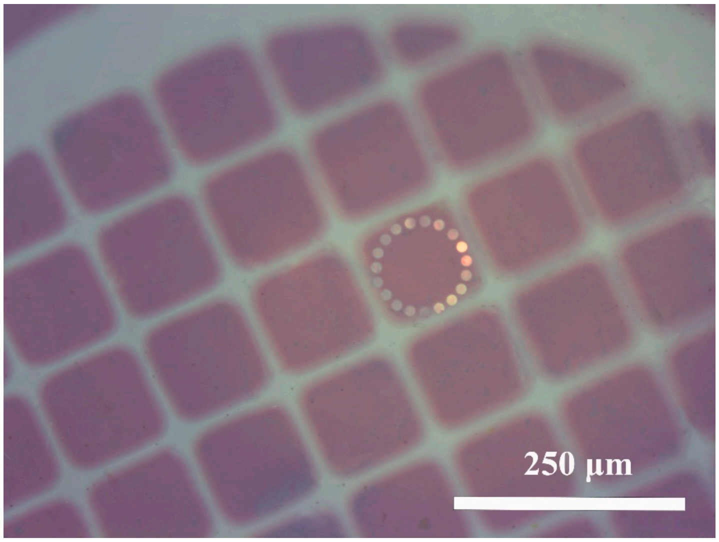

Figure S1. The microscope image of PEN/Au-Ag NPs sample. Aureole circled area was the test region.

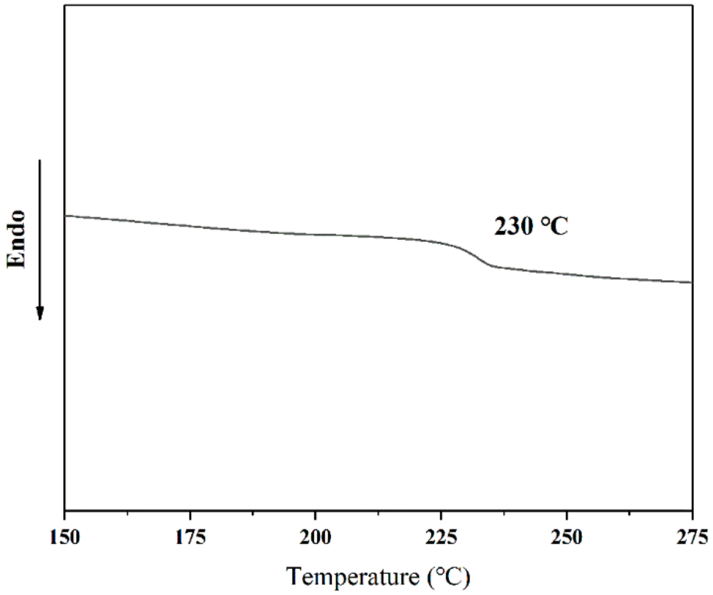

Figure S2. DSC spectrum of employed PEN.

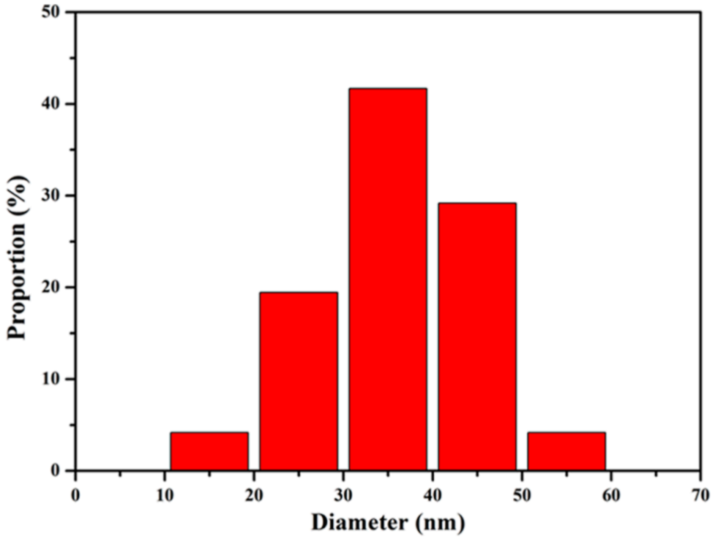

Figure S3. The histograms of the particle size distribution according to the SEM result of PEN/Au-Ag NPs annealing at 290 ^{o}C.

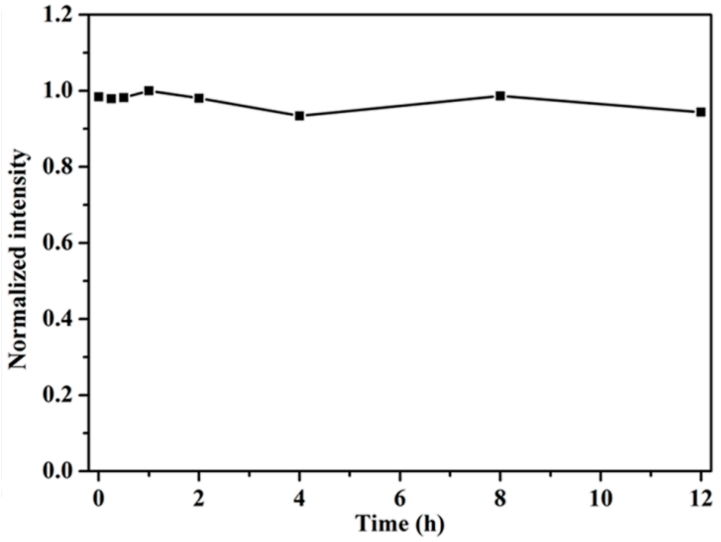

Figure S4. The normalized intensity of PEN/Au-Ag NPs (annealing at 290 ^{o}C) suffered from absolute ethanol for different times.

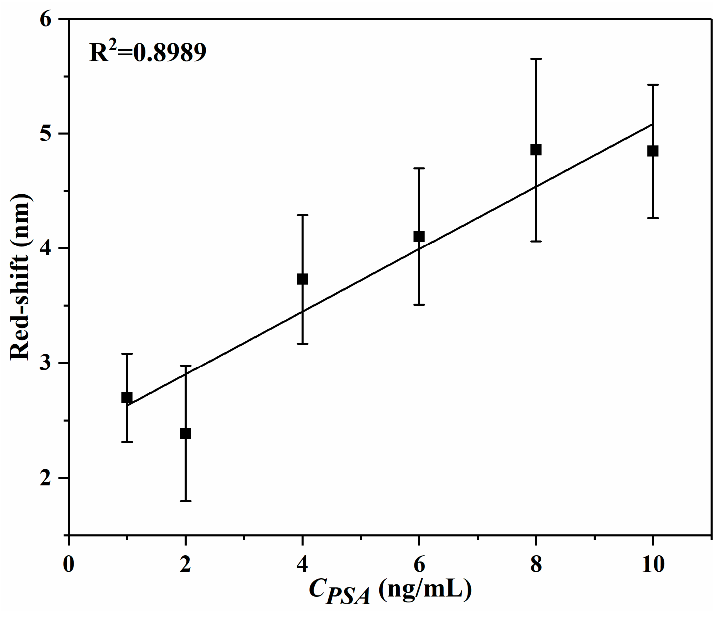

Figure S5. The dose dependent responses to PSA antigen of different concentrations (1 to 10 ng/mL) and the corresponding linear fitting curve.
